# Genomic Characterization, Antimicrobial Resistance Profiles, and *tetA* Nucleotide Substitutions of *Escherichia coli* Isolated from Healthy Dogs in Thailand

**DOI:** 10.3390/ani16132023

**Published:** 2026-07-02

**Authors:** Ravisa Warin, Naparat Suttidate, Wanna Suriyasathaporn, Witaya Suriyasathaporn, Dethaloun Meunsene, Ratchadaporn Boripun

**Affiliations:** 1 Akkhraratchakumari Veterinary College, Walailak University, Nakhon Si Thammarat 80160, Thailand; ravisa.wa@wu.ac.th (R.W.); naparat.st@mail.wu.ac.th (N.S.); 2 One Health Research Center, Walailak University, Nakhon Si Thammarat 80160, Thailand; 3 Faculty of Veterinary Medicine, Chiang Mai University, Chiang Mai 50100, Thailand; wanna.suri@cmu.ac.th (W.S.); suriyasathaporn.witaya.y3@f.mail.nagoya-u.ac.jp (W.S.); 4 Research Center of Producing and Development of Products and Innovations for Animal Health and Production, Chiang Mai University, Chiang Mai 50100, Thailand; 5 Oversea Campus, Asian Satellite Campuses Institute, Nagoya University, Nagoya 464-8601, Japan; 6 Department of Veterinary Medicine, Faculty of Agriculture, National University of Laos, Vientiane 01170, Laos

**Keywords:** antimicrobial resistance, *Escherichia coli*, dog, nucleotide substitutions

## Abstract

Antimicrobial resistance (AMR) in *Escherichia coli* (*E. coli*) from companion dogs is an emerging One Health concern because dogs may serve as asymptomatic reservoirs of multidrug-resistant (MDR) and potentially pathogenic bacteria that can be transmitted to humans. This study aimed to determine antimicrobial susceptibility profiles, characterize antimicrobial resistance and virulence associated genes, and identify nucleotide substitutions in resistance determinants of *E. coli* isolated from healthy dogs in Thailand. Fecal samples (*n* = 200; 100 samples per province) were collected from healthy dogs attending a total of 50 small animal clinics located in Nakhon Si Thammarat and Chiang Mai provinces between January and March 2026. *E. coli* isolates were identified by culture and polymerase chain reaction (PCR) confirmation. Antimicrobial susceptibility was evaluated against 12 antimicrobial agents using the disk diffusion method according to CLSI guidelines. Thirty randomly selected isolates were further screened for resistance and virulence-associated genes by PCR, and the *tetA* gene was sequenced to identify nucleotide substitutions. Differences in gene distributions between provinces were assessed using Fisher’s exact test. A total of 66 *E. coli* isolates were recovered. High resistance rates were observed for ampicillin (100%), piperacillin (84.85%), ceftriaxone (60.61%), tetracycline (56.06%), and aztreonam (46.97%). Multidrug resistance was identified in 47% of the isolates. The 30 isolates exhibiting the highest levels of antimicrobial resistance were selected for molecular characterization. Among these, the β-lactam resistance gene *bla*_TEM_ was significantly more prevalent in Nakhon Si Thammarat than in Chiang Mai (60% vs. 0%; *p* = 0.022). The virulence gene *stx2* was also detected significantly more frequently in isolates from Nakhon Si Thammarat (93.33% vs. 26.67%; *p* < 0.001). Sequence analysis of *tetA* revealed multiple nucleotide substitutions in two isolates, suggesting ongoing genetic variation within tetracycline resistance determinants that may contribute to the evolution and persistence of antimicrobial resistance. These findings demonstrate that healthy dogs in Thailand can act as reservoirs of MDR and potentially virulent *E. coli*. The observed geographic variation in resistance and virulence gene distributions highlights the importance of antimicrobial stewardship and continuous molecular surveillance in companion animals within a One Health framework.

## Simple Summary

Antibiotic resistance is a growing health problem because bacteria that resist treatment can spread between animals, humans, and the environment. Dogs live closely with humans and can carry resistant bacteria in their intestines without appearing sick. This study examined whether healthy dogs in two provinces of Thailand (Nakhon Si Thammarat and Chiang Mai) carry resistant *Escherichia coli* (*E. coli*), a common gut bacterium that can sometimes cause disease. We collected 200 fecal samples between January and March 2026 and identified 66 *E. coli* isolates. Many isolates were resistant to commonly used antibiotics, especially those in the penicillin group and tetracycline, and most were resistant to multiple types of antibiotics at the same time. In a smaller set of isolates, we found genes that help bacteria resist penicillin and tetracycline, with one important resistance gene being more common in Nakhon Si Thammarat. We also detected genes linked to the ability to cause severe intestinal illness, particularly one toxin gene that was much more frequent in Nakhon Si Thammarat. These results show that healthy dogs can act as hidden carriers of resistant and potentially harmful bacteria, supporting routine monitoring and careful antibiotic use in pet healthcare to protect both animal and human health.

## 1. Introduction

Antimicrobial resistance (AMR) is recognized as a major global public health crisis, threatening the effective prevention and treatment of infectious diseases in humans and a wide range of animal species, including livestock, companion animals, and wildlife [1,2]. Gram-negative bacteria, particularly *E. coli*, are among the most important contributors due to their remarkable ability to acquire and disseminate antibiotic resistance genes (ARGs) [3]. Although *E. coli* is a normal commensal inhabitant of the intestinal tract of humans and various animal species, it is an important opportunistic pathogen responsible for gastrointestinal, urinary tract, and systemic infections [4]. Companion animals, especially dogs, have been increasingly identified as potential reservoirs of the antimicrobial-resistant *E. coli*, raising concerns regarding to zoonotic transmission and the One Health interface [5,6]. Close contact between dogs and their owners facilitates the exchange of bacteria and resistance determinants, contributing to the dissemination of MDR strains in household and clinical environments [7,8]. Even clinically healthy dogs may asymptomatically harbor MDR *E. coli* in their gastrointestinal tract, serving as silent carriers of resistance genes [9].

The development of antimicrobial resistance in *E. coli* is largely driven by horizontal gene transfer mediated by plasmids, transposons, and integrons [10,11]. Among the most prevalent resistance determinants are *β*-lactamase genes, such as *bla*_TEM_, which confer resistance to penicillin and extended-spectrum cephalosporins [12,13]. Extended-spectrum *β*-lactamase (ESBL) producing *E. coli* have been reported in companion animals worldwide [14]. Tetracycline resistance is also commonly detected, and frequently associated with the efflux pump genes, such as *tetA* [15,16]. In addition to the acquisition of resistance genes, nucleotide substitutions within these genes may alter enzyme activity, expand substrate specificity, or enhance resistance phenotypes [17]. Molecular characterization and sequencing-based analyses therefore provide critical insights into the genetic diversity and evolutionary dynamics of resistance determinants circulating among diverse animal species, including livestock, companion animals, and wildlife.

Thailand has reported an increasing prevalence of antimicrobial resistance in both humans and various animal species [18,19,20]. However, there is limited data regarding to the molecular characterization and nucleotide substitution patterns of ARGs in MDR *E. coli* isolated from healthy dogs, especially the genetic diversity at different geographical regions. Surveillance of resistance genes in dogs is essential for guiding antimicrobial stewardship programs and mitigating the spread of resistance within the One Health framework [21,22].

To contribute to a better understanding of the molecular epidemiology of MDR *E. coli* in dogs and the evolution of resistance determinants in veterinary settings, this study aimed to (i) determine the antimicrobial susceptibility profiles of *E. coli* isolated from healthy dogs in Thailand, (ii) identify virulence-associated and antimicrobial resistance genes, and (iii) characterize nucleotide substitutions in selected resistance genes using sequencing analysis.

## 2. Materials and Methods

### 2.1. Sample Collection

The sample size was determined using a formula for estimating true prevalence available from Epitools (https://epitools.ausvet.com.au). Due to variation in the reported prevalence of *E. coli*, an expected prevalence of 34.9% was selected for the calculation [22]. The calculation assumed a test sensitivity of 95%, specificity of 90%, desired precision of 10%, and a confidence level of 90%. A total of 200 fecal samples were collected from clinically healthy dogs at small animal clinics in Nakhon Si Thammarat province (*n* = 100) and Chiang Mai province (*n* = 100), Thailand, between January and March 2026. All samples were swabbed in a sterile loop and transported in the ice boxes to the laboratory within one hour for microbiological analysis.

### 2.2. Bacterial Isolation and Identification

The isolation and identification of *E. coli* from fecal samples were performed following a previously described protocol [23]. In brief, all fecal cotton swabs were enriched in 225 mL of alkaline peptone water (Oxoid, Hampshire, UK) and incubated at 37 °C for 24 h. Subsequently, a loopful of the enriched suspension was streaked onto MacConkey agar (Oxoid, Hampshire, UK) and incubated at 37 °C for another 24 h. Pink colonies indicative of lactose fermentation, presumptively *E. coli*, were selected and subcultured on Eosin Methylene Blue (EMB) agar (HiMedia Laboratories, Mumbai, India), followed by incubation at 37 °C for 24 h. Colonies exhibiting a metallic green sheen characteristic of *E. coli* were re-streaked onto Tryptic Soy Agar (TSA) (HiMedia Laboratories, Mumbai, India) and subjected to further confirmation using polymerase chain reaction (PCR).

### 2.3. Antibiotic Susceptibility Testing

Antibiotic susceptibility of the isolates was evaluated using the disc diffusion method. Briefly, 3–5 colonies grown on Mueller Hinton Agar (MHA) (HiMedia Laboratories, Mumbai, India) were suspended in 0.85% normal saline to achieve a turbidity equivalent to the 0.5 McFarland standard. The following antimicrobial agents were tested: ampicillin (AMP), amoxicillin–clavulanic acid (AMC), piperacillin (PRL), ceftriaxone (CRO), aztreonam (ATM), imipenem (IPM), amikacin (AK), gentamicin (CN), enrofloxacin (ENR), tetracycline (TE), chloramphenicol (C), and streptomycin (STR). Inhibition zone diameters were measured and interpreted according to Clinical and Laboratory Standards Institute (CLSI, 2019) guidelines [24]. Isolates resistant to three or more antimicrobial classes were classified as MDR.

### 2.4. Molecular Assessment

**Figure 1:**
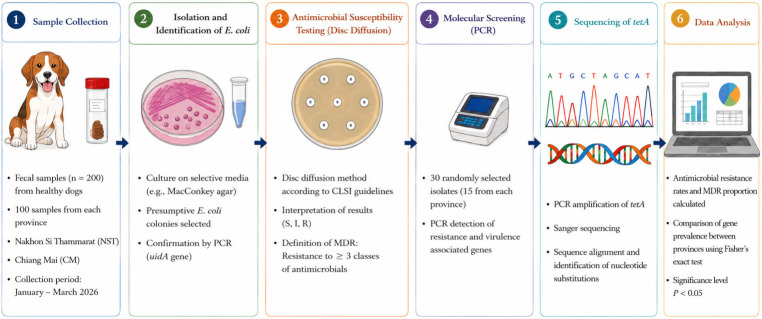
Experimental workflow for bacterial identification, antimicrobial susceptibility testing and molecular characterization of *E. coli*.

Of the 66 *E. coli* isolates recovered, the 30 isolates exhibiting the highest levels of antimicrobial resistance (i.e., resistant to the greatest number of antimicrobial classes) were selected for molecular characterization. This selection was based on the clinical relevance of multidrug-resistant isolates and was also limited by available research funding and project timeline constraints. Selected isolates were subjected to polymerase chain reaction (PCR) for the detection of virulence-associated pathotype genes and antimicrobial resistance genes using specific primers (Table 1), as previously described [25,26,27,28,29,30]. Additionally, sequence analysis of the tetracycline resistance gene (*tetA*) was performed to investigate point mutations that may contribute to antimicrobial resistance evolution. Briefly, 2–3 colonies from each isolate were suspended in 1 mL of distilled water, boiled at 100 °C for 10 min, and centrifuged at 1000 rpm for 5 min. Two microliters of the supernatant was added to a 13 µL PCR Master Mix containing 0.5 µL of 10 mM dNTPs, 2.5 µL of 10× Taq buffer with MgSO_4_, 1 µL of 2.5 mM MgCl_2_, 0.3 µL of Taq DNA polymerase (5 U/µL), 0.2 µL of dimethyl sulfoxide (DMSO), 1 µL each of forward and reverse primers, and 16.5 µL of distilled water. PCR mixture preparation was optimized for each primer set. For resistance gene detection, the amplification protocol consisted of an initial denaturation at 95 °C for 5 min, followed by 30 cycles of denaturation at 95 °C for 35 s, annealing at primer-specific temperatures for 45 s, extension at 52–72 °C for 1 min, and a final extension at 72 °C for 5 min [24,25,26,27,28,29]. For pathotype gene amplification, the PCR conditions included an initial denaturation at 95 °C for 5 min, followed by 35 cycles of denaturation at 95 °C for 30–45 s, annealing at 56–61 °C for 30 s, and a final extension at 72 °C for 5 min [30]. PCR products (5 µL) were analyzed by electrophoresis on a 1.5% agarose gel in 0.5× TBE buffer at 135 V for 40 min. DNA bands were visualized under ultraviolet illumination. Positive PCR products were purified and sequenced using Sanger sequencing (Macrogen^®^, Seoul, Republic of Korea). The overall study design and experimental workflow used for bacterial identification, antimicrobial susceptibility testing, and molecular characterization of *E. coli* are summarized in Figure 1.

### 2.5. Analysis of DNA Sequences

DNA sequences were trimmed using Chromatogram Explorer Lite version 5.0.2 (Heracle BioSoft SRL, Pitesti, Romania; http://www.dnabaser.com). The trimmed chromatograms were further edited with SnapGene^®^ Viewer version 5.3.2 (GSL Biotech LLC, San Diego, CA, USA; https://www.snapgene.com/snapgene-viewer, accessed on 20 March 2026). Edited sequences were analyzed using the BLAST tool from the National Center for Biotechnology Information (NCBI, Bethesda, MD, USA; https://blast.ncbi.nlm.nih.gov/Blast.cgi, accessed on 20 March 2026) to confirm identity. Confirmed *E. coli* sequences were then subjected to polymorphism analysis based on the number of variable sites using DNA Sequence Polymorphism software (DnaSP) version 6.12.03 (University of Barcelona, Barcelona, Spain; http://www.ub.edu/dnasp; accessed on 20 March 2026). Nucleotide substitutions in resistance genes were identified by comparing sample sequences with reference sequences from public databases.

### 2.6. Statistical Analysis

Statistical analyses were performed using *R* software (version 4.0.2). Descriptive statistics were used to summarize the prevalence of *E. coli*, antimicrobial susceptibility, MDR patterns, and gene distributions. Differences in *E. coli* prevalence between provinces were assessed using the chi-square test, with prevalence ratios (PR) and 95% confidence intervals (95% *CI*) calculated. Comparisons of antimicrobial resistance and virulence-associated genes were performed using Fisher’s exact test (two-tailed), based on positive and negative isolates. Gene-level analysis was applied where isolate-level data were unavailable. Variables without variation were excluded. A *p*-value < 0.05 was considered a statistical significance.

## 3. Results

### 3.1. Identification of E. coli

A total of 200 samples (100 samples per province) were analyzed for the detection of *E. coli*. The overall prevalence was higher in Nakhon Si Thammarat province (38.0%, 38/100) than in Chiang Mai province (28.0%, 28/100) (Table 2). Although the prevalence was higher in Nakhon Si Thammarat province (38%) than in Chiang Mai province (28%), the difference was not statistically significant (Chi-square test, *p* = 0.13) (Table 2).

### 3.2. Antimicrobial Resistance Profiles

**Figure 2:**
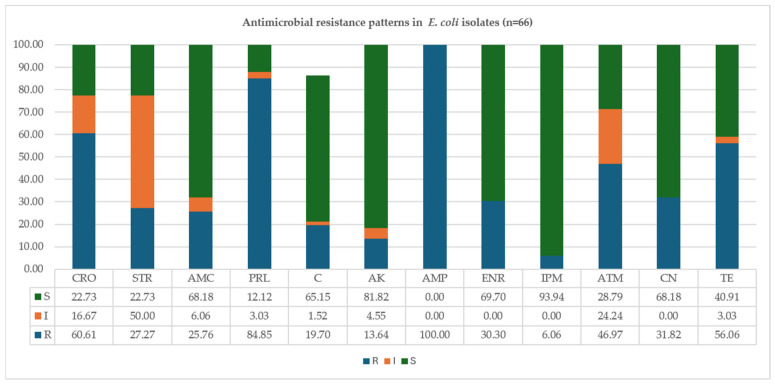
Antimicrobial resistance patterns in *E. coli* isolates (*n* = 66). Resistant (R), intermediate (I), and susceptible (S) isolates are shown in blue, orange, and gray, respectively. The antimicrobial agents tested were: CRO (ceftriaxone), STR (streptomycin), AMC (amoxicillin–clavulanic acid), PRL (piperacillin), C (chloramphenicol), AK (amikacin), AMP (ampicillin), ENR (enrofloxacin), IPM (imipenem), ATM (aztreonam), CN (gentamicin), and TE (tetracycline).

A total of 66 *E. coli* isolates were tested for the susceptibility for 12 antimicrobial agents. The distributions of resistant (R), intermediate (I), and susceptible (S) isolates are presented in Figure 2. For resistance, all isolates showed the highest levels of resistance to ampicillin (AMP) (100%). They also showed high levels of resistance to piperacillin (PRL) (84.85%), ceftriaxone (CRO) (60.61%), tetracycline (TE) (56.06%), and aztreonam (ATM) (46.97%). Moreover, they showed moderate resistance levels to gentamicin (CN) (31.82%), enrofloxacin (ENR) (30.30%), streptomycin (STR) (27.27%), and amoxicillin–clavulanic acid (AMC) (25.76%). Lastly, they showed the low levels of resistance to chloramphenicol (C) (19.70%), amikacin (AK) (13.64%), and imipenem (IPM) (6.06%).

For susceptibility, IPM demonstrated the highest susceptible rate (93.94%), followed by AK (81.82%), ENR (69.70%), AMC (68.18%), CN (68.18%), and C (65.15%), respectively. Moderate susceptibility rates were observed in TE (40.91%) and ATM (28.79%), whereas low susceptibility rates were detected for CRO (22.73%), S (22.73%), and PRL (12.12%). Notably, no isolate was susceptible to AMP. For I isolates, high levels were detected in STR (50.00%) and ATM (24.24%), while CRO, AMC, PRL, C, AK, and TE showed moderate levels. No intermediate isolate was detected in AMP, ENR, IPM, or CN.

### 3.3. Multidrug Resistance Profile

A total of 31 isolates (47.0% of 66 isolates) exhibited multidrug resistance (MDR), defined as resistance to three or more antimicrobial classes, comprising 18 distinct resistance profiles. The most common MDR profiles were STR–PRL–C–AMP–CN–TE (*n* = 5), CRO–STR–AMC–PRL–AMP–ENR–IPM–ATM–CN–TE (*n* = 3), and CRO–AMC–PRL–C–AK–AMP–ENR–CN–TE (*n* = 2). Among MDR isolates, resistance to four antimicrobial classes was most prevalent (*n* = 20; 64.5%), followed by resistance to three classes (*n* = 6; 19.4%), and five classes (*n* = 5; 16.1%). Beta-lactam resistance was present in all MDR profiles and was frequently co-occurring with resistance to aminoglycosides, fluoroquinolones, tetracycline, and phenicols. Notably, four isolates (*n* = 4; CRO–AMC–PRL–AMP–ENR–IPM–ATM–CN–TE, *n* = 1; CRO–STR–AMC–PRL–AMP–ENR–IPM–ATM–CN–TE, *n* = 3) demonstrated resistance to imipenem, a carbapenem, suggesting an emerging reduced susceptibility to last-resort antibiotics (Table 3).

### 3.4. Detection of Antibiotic Resistance Genes

Among the 66 *E. coli* isolates recovered, the 30 isolates exhibiting the highest levels of antimicrobial resistance (15 isolates from Nakhon Si Thammarat Province and 15 isolates from Chiang Mai Province) were selected for molecular characterization by PCR. This selection was based on the clinical relevance of multidrug-resistant (MDR) isolates. Among the selected isolates, virulence genes associated with Shiga toxin-producing *E. coli* (STEC) and enteropathogenic *E. coli* (EPEC) were detected at varying frequencies between the two provinces. In contrast, genes associated with enterotoxigenic *E. coli* (ETEC), enteroinvasive *E. coli* (EIEC), and enteroaggregative *E. coli* (EAEC) were not detected in any isolate. No significant difference in the distribution of virulence genes was observed between the provinces (*p* = 0.619).

Regarding β-lactam resistance genes, *bla*_TEM_ was detected in 60% of isolates from Nakhon Si Thammarat Province but was absent in isolates from Chiang Mai Province (0%). The *bla*_CTX_ gene was identified in 66.67% and 40.00% of isolates from Nakhon Si Thammarat and Chiang Mai Provinces, respectively, whereas *bla*_OXA_ was detected in 20.00% and 26.67% of isolates, respectively. The *bla*_SHV_ gene was not detected in any isolate. Overall, a significant difference in the distribution of β-lactam resistance genes was observed between the two provinces (*p* = 0.022). All isolates from both provinces carried the fluoroquinolone resistance gene *parC* (100%; *p* = 1.000). For phenicol resistance, the *cmlA* gene was detected in 53.33% of isolates from Nakhon Si Thammarat Province but was not detected in any isolate from Chiang Mai Province, whereas the chloramphenicol acetyltransferase (*cat*) gene was not detected in either group. Overall, a significant difference in the distribution of phenicol resistance genes was observed between the two provinces (*p* = 0.005). Among aminoglycoside resistance genes, *aac(3)-IV* was the most prevalent, being detected in 53.33% and 73.33% of isolates from Nakhon Si Thammarat and Chiang Mai Provinces, respectively. This was followed by *aac(3)-I*, which was detected in 46.67% and 13.33% of isolates, respectively, while *aphA-1* was identified in 20.00% of isolates from both provinces. However, no significant difference in the distribution of aminoglycoside resistance genes was observed between the two provinces (*p* = 0.828) (Table 4).

### 3.5. Detection of Virulence Associated Pathotype Genes

Among the 30 isolates (15 from Nakhon Si Thammarat province and 15 from Chiang Mai province), the detection rates of the virulence-associated gene characteristics of STEC and EPEC varied. However, ETEC-, EIEC-, and EAEC-associated genes were not identified in any isolate. The *stx-2* gene (STEC) showed significantly higher prevalence in Nakhon Si Thammarat province isolates (14/15; 93.33%) than those from Chiang Mai province (4/15; 26.67%) (Fisher’s exact test, two-tailed; *p* < 0.001). In contrast, the *stx-1* gene was not detected in either group. For EPEC-associated genes, *eae* was detected in all isolates from Nakhon Si Thammarat province (15/15; 100%) and in 12 of 15 isolates (80%) from Chiang Mai province. However, there was no statistically significant difference between provinces (*p* = 0.224). The *bfp* gene was not detected in any isolate. None of the isolates carried ETEC-associated genes (*lt*, *stII*), EIEC-associated genes (*virF*, *ipaH*), or the EAEC-associated gene (*aafII*). Statistical comparisons were only performed for genes with variability between groups (*stx-2* and *eae*), as genes absent in both groups (0%) could not be analyzed due to a lack of variability (Table 5).

### 3.6. Nucleotide Substitution of the Resistant Genes

**Figure 3:**
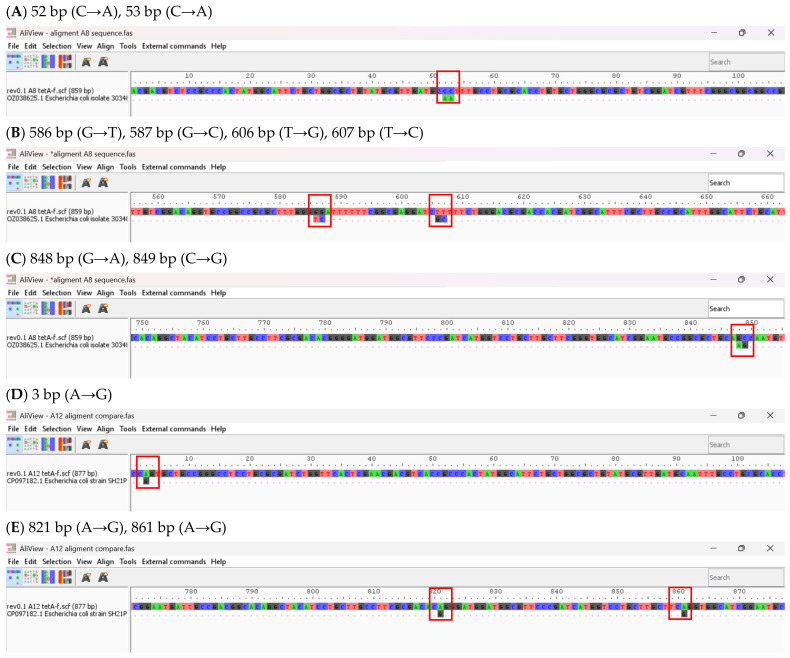
Nucleotide substitution of *tetA* gene in *E*. *coli* Ecom_008 (**A**–**C**), *tetA* gene in Ecom_012 (**D**,**E**).

The point mutation analysis of the tetracycline resistance gene (*tetA*) was performed in the *E. coli* isolates Ecom_008 and Ecom_012 (Table 6 and Figure 3). In isolate Ecom_008, the *tetA* gene sequence had a length of 859 bp. Eight-point mutations were identified. Two adjacent substitutions occurred at the positions 52 bp and 53 bp (C→A). Additional paired substitutions were detected at the positions 586 bp (G→T) and 587 bp (G→C), as well as at the positions 606 bp (T→G) and 607 bp (T→C). Two further mutations were observed near the 3′ end of the gene at positions 848 bp (G→A) and 849 bp (C→G). These mutations were distributed across the gene and included multiple consecutive nucleotide substitutions. In isolate Ecom_012, the *tetA* gene sequence was 877 bp in length. Three-point mutations were detected at positions 3 bp, 821 bp, and 861 bp, all involving A→G transitions (Table 6).

## 4. Discussion

The prevalence of *E. coli* was higher in dogs from Nakhon Si Thammarat province (38%) than in Chiang Mai province (28%); however, the difference was not statistically significant (*p* = 0.13). This observed variation may reflect geographical differences, although further studies with larger sample sizes are needed to confirm this finding. As *E. coli* is a common commensal organism in the intestinal tract of dogs, its detection in fecal samples reflects a colonization rather than an infection. One limitation of this study is that only lactose-fermenting colonies were selected for *E. coli* isolation. Therefore, atypical non-lactose-fermenting strains carrying antimicrobial resistance or virulence genes may have been overlooked, potentially leading to an underestimation of the diversity of resistant *E. coli* isolates. However, *E. coli* detection remains an important indicator for antimicrobial resistance surveillance. Although a higher prevalence of *E. coli* was observed in Nakhon Si Thammarat than in Chiang Mai, the difference was not statistically significant. Factors such as dog management practices, living conditions, feeding practices, veterinary care, and antimicrobial exposure have been reported to influence the intestinal microbiota and the carriage of *E. coli* in companion animals and may contribute to variations in prevalence across populations. Previous studies show that antimicrobial use in companion animals is a key driver of the selection and persistence of resistant *E. coli* strains [31]. In addition, close contact between dogs and humans facilitates the potential exchange of *E. coli*, including antimicrobial-resistant strains. Several studies report that dogs can act as reservoirs of resistant bacteria, with possible transmission between pets and their owners [32,33]. Moreover, certain lineages of *E. coli* identified in companion animals overlap with those found in humans, highlighting the importance of public health surveillance of *E. coli* in dogs [34,35]. The detection of *E. coli* in a substantial proportion of fecal samples underscores the importance of continued surveillance in companion dogs. Monitoring *E. coli* carriage in dogs can provide valuable baseline data for future studies on antimicrobial resistance and contribute to a better understanding of the epidemiology of zoonotic bacteria. Phenotypic antimicrobial susceptibility testing revealed extensive resistance to *β*-lactams, including ampicillin, piperacillin, and ceftriaxone, consistent with the global trend of *β*-lactam resistance in animal-associated *E. coli* [36,37]. This pattern likely reflects selective pressure from prolonged veterinary use of penicillins and cephalosporins, facilitating the emergence and persistence of resistant strains [38]. Tetracycline resistance showed high prevalence, primarily mediated by *tetA*, *tetB*, and *tetC*. Similarly, many studies demonstrate that the conjugative plasmids play a major role in the horizontal transfer of tetracycline resistance genes across bacterial populations [39,40,41,42,43,44,45,46,47,48,49,50,51]. Resistance to aminoglycosides and fluoroquinolones was moderate. Fluoroquinolone resistance is a particular concern due to its critical role in both humans and veterinary medicine, with a common association with chromosomal mutations (e.g., *parC*) and plasmid-mediated determinants [42,52,53]. Conversely, carbapenems and amikacin exhibited low resistance rates, reflecting their limited use in veterinary medicine and their preservation as last resort antimicrobials in human medicine [47,49]. A significant proportion of isolates exhibited MDR, with 18 distinct resistance profiles. The co-occurrence of multiple resistance genes, often located on mobile genetic elements such as plasmids, integrons, and transposons, underscores the potential for horizontal gene transfer and amplification of resistance within microbial communities [47]. Notably, the prevalence of *β*-lactamase genes (*bla*_TEM_, *bla*_CTX_, *bla*_OXA_) suggests the circulation of extended-spectrum *β*-lactamase (ESBL)-producing *E. coli*, which is of major public health concern due to its capacity to hydrolyze a broad range of *β*-lactam antibiotics [54,55].

Virulence gene analysis revealed that the presence of Shiga toxin-producing *E. coli* (STEC) and enteropathogenic *E. coli* (EPEC), with the detection of *stx2* and *eae* genes. These pathotypes are associated with severe human infections, including hemorrhagic colitis, and hemolytic uremic syndrome, emphasizing the zoonotic potential of these isolates [56,57,58,59,60]. Dogs may act as reservoirs of pathogenic and antimicrobial-resistant *E. coli*, suggesting a potential zoonotic risk through close contact and environmental contamination [58,59,61]. The detection of *stx2*- and *eae*-positive isolates is of particular public health concern because these virulence factors are associated with severe gastrointestinal disease in humans. The coexistence of virulence determinants and resistance to commonly used first-line antimicrobials, particularly AMP, may further complicate treatment options if zoonotic transmission occurs. As companion dogs frequently live in close contact with humans, asymptomatic carriage of STEC and atypical EPEC may facilitate household and environmental dissemination of potentially pathogenic and MDR *E. coli* strains.

The absence of the *bfp* gene suggests the predominance of atypical EPEC strains, which are increasingly recognized in both animal and environmental sources [62]. From a One Health perspective, the coexistence of antimicrobial resistance and virulence determinants in animal-associated *E. coli* highlights the interconnected risks across humans, animals, and the environment. Resistant strains in dogs may spread through close human contact and environmental dissemination (e.g., water or soil contamination). This reinforces the need for integrated surveillance strategies and an antimicrobial stewardship across sectors to mitigate zoonotic transmission and preserve public health [61,62,63,64,65,66,67,68,69,70]. Finally, mutation analysis of the *tetA* gene demonstrated nucleotide diversity among isolates, suggesting adaptive evolution under selective antimicrobial pressure. Such genetic variation within resistance determinants may influence persistence and transmission dynamics in microbial populations [63,64].

This study has several limitations. First, only multidrug-resistant isolates were selected for molecular characterization, which may not fully represent the diversity of antimicrobial resistance genes present in the overall *E. coli* population. Second, samples were collected from only two provinces in Thailand, limiting the generalizability of the findings to other geographic regions. Third, the cross-sectional design provides a snapshot of antimicrobial resistance patterns and does not allow for assessment of temporal changes. Fourth, only lactose-fermenting colonies were selected for E. coli isolation; therefore, atypical non-lactose-fermenting strains carrying antimicrobial resistance or virulence genes may have been overlooked. In addition, further characterization of carbapenem-resistant isolates, including carbapenemase gene detection and lateral flow immunoassay testing, was not performed because these analyses were beyond the scope and available resources of the current study. Finally, isolate-level co-occurrence data for antimicrobial resistance genes were unavailable, requiring gene-level aggregation for statistical analyses within antimicrobial classes. Therefore, the findings should be interpreted with appropriate caution. Future studies involving larger sample sizes, broader geographic coverage, longitudinal sampling, and comprehensive molecular characterization are warranted to better understand the epidemiology and dissemination of antimicrobial-resistant *E. coli* in companion dogs.

Overall, these findings underscore the role of dogs as reservoirs of MDR and potentially pathogenic *E. coli*. Continuous surveillance and coordinated One Health interventions are critical to mitigating the spread of AMR and pathogenic *E. coli* along the human–animal–environment interface [65,66,67,68,69,70].

## 5. Conclusions

This study demonstrated that healthy dogs in Thailand can harbor MDR *E. coli* carrying antimicrobial resistance and virulence-associated genes. High resistance rates to β-lactam antibiotics and the detection of STEC- and EPEC-associated genes highlight the potential public health significance of these isolates. The findings emphasize the importance of continuous AMR surveillance, prudent antimicrobial use in veterinary medicine, and a One Health approach to reduce the spread of resistant and potentially zoonotic *E. coli* at the human–animal interface. Further genomic studies with larger sample sizes are needed to better understand the transmission dynamics and evolution of antimicrobial resistance in dogs.

## Figures and Tables

**Table 1:** Primers used for the amplification of resistance genes and pathotypes.

Detection	Target Genes	Primer Sequences	Product Size (bp)	References
Tetracycline-resistant genes	*tetA*	F: 5′-GTAATTCTGAGCACTGTCGC-3′	965	[24]
		R: 5′-CTGCCTGGACAACATTGCTT-3′		
	*tetB*	F: 5′-CTCAGTATTCCAAGCCTTTG-3′	414	
		R: 5′-ACTCCCCTGAGCTTGAGGGG-3′		
	*tetC*	F: 5′-CCTCCTGCGGGATATCGTCC-3′	505	
		R: 5′-GGTTGAAGGCTCTCAAGGGC-3′		
Beta-lactam-resistant genes	*bla*_TEM_	F: 5′-CGCCGCATACACTATTCTCAGAATGA-3′	445	[25]
		R: 5′-ACGCTCACCGGCTCCAGATTTAT-3′		
	*bla*_SHV_	F: 5′-ATGCGTTATATTCGCCTGTG-3′	747	
		R: 5′-TGCTTTGTTATTCGGGCCAA-3′		
	*bla*_CTX_	F: 5′-GAATTAGAGCGGCAGTCGGG-3′	588	[26]
		R: 5′-GATGGCGACGCTACCCC-3′		
	*bla*_OXA_	F: 5′-GCAGCGCCAGTGCATCAAC-3′	198	[27]
		R: 5′-CCGCATCAAATGCCATAAGTG-3′		
Fluoroquinolones Phenicols	*parC*	F: 5′-GCCTTGCGCTACATGAATTT-3′	311	[28]
		R: 5′-CAACGAAATCGACCGTCTCT-3′		
	*cat1*	F: 5′-AGTTGCTCAATGTACCTATAACC-3′	547	[29]
		R: 5′-TTGTAATTCATTAAGCATTCTGCC-3′		
	*cmlA*	F: 5′-CCGCCACGGTGTTGTTGTTATC-3′	698	
		R: 5′-CACCTTGCCTGCCCATCATTAG-3′		
Aminoglycosides	*aac(3)-I*	F: 5′-ACCTACTCCCAACATCAGCC-3′	157	
		R: 5′-ATATAGATCTCACTACGCGC-3′		
	*aphA-1*	F: 5′-ATGGGCTCGCGATAATGTC-3′	600	
		R: 5′-CTCACCGAGGCAGTTCCAT-3′		
	*aac(3)-IV*	F: 5′-CTTCAGGATGGCAAGTTGGT-3′	286	
		R: 5′-TCATCTCGTTCTCCGCTCAT-3′		
STEC	*stx-1*	F: 5′-CAGTTAATGTGGTGGCGAAGG-3′	348	[30]
		R 5′-CACCAGACAATGTAACCGCTG-3′		
	*stx-2*	F: 5′-ATCCTATTCCCGGGAGTTTACG-3′	584	
		R: 5′-GCGTCATCGTATACACAGGAGC-3′		
EPEC	*bfp*	F: 5′-GGAAGTCAAATTCATGGGGGTAT-3′	300	
		R: 5′-GGAATCAGACGCAGACTGGTAGT-3′		
	*eae*	F: 5′-TCAATGCAGTTCCGTTATCAGTT-3′	482	
		R: 5′-GTAAAGTCCGTTACCCCAACCTG-3′		
ETEC	*It*	F: 5′-GCACACGGAGCTCCTCAGTC-3′	218	
		R: 5′-TCCTTCATCCTTTCAATGGCTTT-3′		
	*stII*	F: 5′-AAAGGAGAGCTTCGTCACATTTT-3′	129	
		R: 5′-AATGTCCGTCTTGCGTTAGGAC-3′		
EIEC	*virF*	F: 5′-AGCTCAGGCAATGAAACTTTGAC-3′	618	
		R: 5′-TGGGCTTGATATTCCGATAAGTC-3′		
	*ipaH*	F: 5′-CTCGGCACGTTTTAATAGTCTGG-3′	933	
		R: 5′-GTGGAGAGCTGAAGTTTCTCTGC-3′		
EAEC	*aafII*	F: 5′-CACAGGCAACTGAAATAAGTCTGG-3′	378	
		R: 5′-ATTCCCATGATGTCAAGCACTTC-3′		

**Table 2:** Prevalence of *E. coli* from Nakhon Si Thammarat and Chiang Mai, Thailand.

Location	Negative (%)	Positive (%)	*p*-Value
Nakhon Si Thammarat (*n* = 100)	62	38	0.13
Chiang Mai (*n* = 100)	72	28	

**Table 3:** MDR profiles of *E. coli* isolates (*n* = 66).

MDR Profiles	Isolates	Drug Class
CRO-STR-AMC-PRL-AMP-ENR-ATM-CN-TE	1	4
CRO-AMC-PRL-C-AK-AMP-ENR-CN-TE	2	4
AMC-PRL-AK-AMP-ENR-CN	3	3
CRO-AMC-PRL-AMP-ENR-IPM-ATM-CN-TE	1	4
AMC-PRL-AK-AMP-ENR-CN-TE	1	4
CRO-AMC-PRL-C-AK-AMP-ENR-ATM-CN-TE	1	4
AMC-PRL-C-AK-AMP-ENR-CN-TE	1	5
AMC-PRL-AMP-ENR-TE	1	3
STR-AMC-PRL-AMP-ENR-TE	1	4
CRO-STR-AMC-PRL-C-AMP-ENR-ATM-TE	1	5
CRO-STR-PRL-C-AMP-ENR-ATM-TE	1	5
STR-PRL-C-AMP-CN-TE	5	4
STR-PRL-AMP-ENR-TE	1	4
CRO-STR-AMC-PRL-AMP-ENR-IPM-ATM-CN-TE	3	4
STR-PRL-AMP-CN-TE	1	3
STR-PRL-AMP-TE	1	3
CRO-STR-PRL-C-AMP-ENR-ATM-TE	1	5
CRO-STR-PRL-AMP-ENR-ATM-CN-TE	1	4
Total	31	

**Table 4:** Distribution of antibiotic resistance genes detected by PCR among multidrug-resistant *E. coli* isolates from Nakhon Si Thammarat Province (*n* = 15) and Chiang Mai Province (*n* = 15).

Drug Class	Primer	Samples (No. of the Isolates)		*p*-Value
		Nakhon Si Thammarat (*n*, %)	Chiang Mai (*n*, %)	
Tetracyclines	*tetA*	7, 46.67%	9, 60%	0.619
	*tetB*	2, 13.33%	0, 0%	
	*tetC*	0, 0%	3, 20%	
Beta-lactams	*bla*_TEM_	9, 60%	0, 0%	0.022
	*bla*_SHV_	0, 0%	0, 0%	
	*bla*_CTX_	10, 66.67%	6, 40%	
	*bla*_OXA_	3, 20%	4, 26.67%	
Fluoroquinolones	*parC*	15, 100%	15, 100%	1
Phenicols	*cat*	0, 0%	0, 0%	0.005
	*cmlA*	8, 53.33%	0, 0%	
Aminoglycosides	*aac(3)-I*	7, 46.67%	2, 13.33%	0.828
	*aphA-1*	3, 20%	3, 20%	
	*aac(3)-IV*	8, 53.33%	11, 73.33%	

**Table 5:** Detection of virulence-associated pathotype genes (*n* = 30).

Detection	Target Genes	Samples (No. of the Isolates)		*p*-Value
		Nakhon Si Thammarat (*n*, %)	Chiang Mai (*n*, %)	
STEC	*stx-1*	0, 0%	0, 0%	
	*stx-2*	14, 93.33%	4, 26.67%	*p* < 0.001
EPEC	*bfp*	0, 0%	0, 0%	
	*eae*	15, 100%	12, 80%	*p* = 0.224
ETEC	*It*	0, 0%	0, 0%	
	*stII*	0, 0%	0, 0%	
EIEC	*virF*	0, 0%	0, 0%	
	*ipaH*	0, 0%	0, 0%	
EAEC	*aafII*	0, 0%	0, 0%	

Note: We calculated *p*-values by Fisher’s exact test (two-tailed), and we compared the number of positive and negative isolates between the two provinces (*n* = 15 each). Only genes (*stx-2* and *eae*) with a variation between groups were tested. Genes with 0% in both groups could not have a calculated *p*-value (no variability).

**Table 6:** Details of the point mutation of each resistant gene.

Isolates Genes	Genes	Length (bp)	Variable	Position
Ecom_008	*tetA*	859	52 bp	C→A
			53 bp	C→A
			586 bp	G→T
			587 bp	G→C
			606 bp	T→G
			607 bp	T→C
			848 bp	G→A
			849 bp	C→G
Ecom_012	*tetA*	877	3 bp	A→G
			821 bp	A→G
			861 bp	A→G

## Data Availability

The data presented in this study are available in the article.
